# Zinc finger protein ZBTB20 expression is increased in hepatocellular carcinoma and associated with poor prognosis

**DOI:** 10.1186/1471-2407-11-271

**Published:** 2011-06-25

**Authors:** Qing Wang, Ye-xiong Tan, Yi-bin Ren, Li-wei Dong, Zhi-fang Xie, Liang Tang, Dan Cao, Wei-ping Zhang, He-ping Hu, Hong-yang Wang

**Affiliations:** 1 International Cooperation Laboratory on Signal Transduction, Eastern Hepatobiliary Surgery Institute, the Second Military Medical University, 225 Changhai Road, Shanghai, 200433, PR China; 2 Department of Pathophysiology, Basic Medicine Institute, Second Military Medical University,800 Xiangyin Road, Shanghai, 200433, PR China; 3 Department of Comprehensive Treatment II, Eastern Hepatobiliary Surgery Institute, the Second Military Medical University, 225 Changhai Road, Shanghai, 200433, PR China

## Abstract

**Background:**

Our previous studies showed that ZBTB20, a new BTB/POZ-domain gene, could negatively regulate α feto-protein and other liver-specific genes, concerning such as bio-transformation, glucose metabolism and the regulation of the somatotropic hormonal axis. The aim of this study is to determine the potential clinical implications of ZBTB20 in hepatocellular carcinoma (HCC).

**Methods:**

Quantitative real-time RT-PCR and Western blot analyses were used to detect expression levels of ZBTB20 in 50 paired HCC tumorous and nontumorous tissues and in 20 normal liver tissues. Moreover, expression of ZBTB20 was assessed by immunohistochemistry of paired tumor and peritumoral liver tissue from 102 patients who had undergone hepatectomy for histologically proven HCC. And its relationship with clinicopathological parameters and prognosis was investigated.

**Results:**

Both messenger RNA and protein expression levels of ZBTB20 were elevated significantly in HCC tissues compared with the paired non-tumor tissues and normal liver tissues. Overexpressed ZBTB20 protein in HCC was significantly associated with vein invasion (*P *= 0.016). Importantly, the recurrence or metastasis rates of HCCs with higher ZBTB20 expression were markedly greater than those of HCCs with lower expression (*P *= 0.003, *P *= 0.00015, respectively). Univariate and multivariate analyses revealed that ZBTB20 overexpression was an independent prognostic factor for HCC. The disease-free survival period and over-all survival period in patients with overexpressed ZBTB20 in HCC was significantly reduced.

**Conclusions:**

The expression of ZBTB20 is increased in HCC and associated with poor prognosis in patients with HCC, implicating ZBTB20 as a candidate prognostic marker in HCC.

## Background

Hepatocellular carcinoma (HCC) is the fifth most-common malignancy in the world and is the third cause of cancer-related death worldwide. The development and progression of HCC is a complicated process involving multiple genes and several transforming steps [1,2]. The exact molecular mechanisms underlying hepatocarcinogenesis have not yet been elucidated. Therefore, searching for new HCC associated molecules may give some clues to study the mechanism of HCC and provide prognostic value in clinical issues.

The BTB/POZ-ZF [Broad complex, Tramtrack, Bric a' brac (BTB) or poxvirus and zinc finger (POZ)-zinc finger] protein family comprises a diverse group of transcription factors. These factors are so named because of a distinct and unique N-terminal BTB/POZ domain and C-terminal DNA-binding zinc finger domains. These proteins have been demonstrated to participate in a wide variety of cellular functions including transcriptional regulation, cellular proliferation, apoptosis, cell morphogenesis, ion channel assembly, and protein degradation through ubiquitination-proteasome system [3-5]. A subset of BTB/POZ proteins have been implicated in human cancer, and they include BCL-6 (B-cell lymphoma 6) [6-9], PLZF (promyelocytic leukemia zinc finger) [10-14], leukemia/lymphoma-related factor (LRF)/Pokemon [15-18], HIC-1 (hypermethylated in cancer-1) [19-23], NAC-1[24-29] and Kaiso [30-33].

ZBTB20 gene, also named DPZF [34], HOF [35], and ZNF288 [36], is a new member of the BTB/POZ-ZF family. It has two isoforms due to the alternative translation initiation, both containing an intact N-terminal BTB domain and a C-terminal zinc finger domain [35]. Zbtb20 is preferentially expressed by hippocampal progenitors, and essential for hippocampal development [37].

In liver, our previous work showed that human ZBTB20 is expressed in fetal liver [34]. In mouse, Zbtb20 is developmentally up-regulated in postnatal liver, and acts as a key transcription repressor of AFP [38]. What's more, ZBTB20 may play an essential role in liver intrinsic functions, possibly through regulating genes such as P450 family members, glucose metabolism and the regulation of the somatotropic hormonal axis [39].

In the present study, we investigated the expression of ZBTB20 in HCC tissues and its potential association with clinicopathological features and post-resectional survival. Our results suggested that ZBTB20 overexpression can be used as an independent marker for the prognosis of patients with HCC.

## Methods

### Patients and liver specimens

Primary HCC tissue sample (*n *= 152) were randomly obtained from patients who underwent routine curative surgery at the Eastern Hepatobiliary Surgery Hospital between 2001 and 2007. The patients were not pretreated with radiotherapy or chemotherapy prior to surgery. Among them, 102 tissue samples were fixed in 10% formalin and embedded in paraffin for immunohistochemical analysis. The other 50 tissue samples were immediately frozen in liquid nitrogen and stored at -80°C after hepatectomy for western blotting and real-time RT-PCR. Table 1 shows the clinicopathological features of these patients. Additionally, normal liver specimens were obtained from 20 patients with hemangiomas of liver who underwent surgery. The 20 cirrhotic liver specimens were obtained from patients who underwent liver biopsy. The diagnoses were confirmed by histopathologic study. Tumor stage was determined according to the 2002 International Union Against Cancer TNM classification system [40]. Tumor differentiation was graded by the Edmondson grading system. Written informed consent was obtained from these patients, and the protocol for this study was approved by the Ethics Committee of the Eastern Hepatobiliary Surgery Hospital.

**Table 1:** Clinicopathological parameters of 152 patients with HCC

Clinicopathological parameters variables	n, range All patients (n = 152)
**Age(years)**	
>50	84
≤50	68
**Gender**	
Male	118
Female	34
**Virus**	
HBV	124
HCV	4
Both	0
None	24
**AFP(ng/ml)**	
≤20	36
>20	116
**Liver cirrhosis**	
Yes	108
No	44
**Pathological stage**	
I	3
II	13
III	131
IV	5
**Tumour size (mm)**	
>50	112
≤50	40
**Tumour multiplicity**	
Solitary	121
Multiple	31
**Vein invasion**	
Absent	127
Present	25
**Tumor encapsulation**	
Complete	65
Uncomplete	34
No	53

### Cells and cell culture

L02, QSG7701, PLC/PRF/5, SMMC7721, HepG2, SK-Hep-1, WRL68, Hep3B, Huh7, HCCCLM3, and HCCC97L cell lines were obtained from American Type Culture Collection (Manassas, VA) or Cell bank of Chinese Academy of Sciences (Shanghai, China). All cells were cultured in DMEM (GiBco BRL, Life Technologies) with 10% fetal bovine serum at 37°C in a humidified 5% CO_2 _atmosphere.

### Immunohistochemistry

Histological diagnoses of tumourous and non-tumourous formalin-fixed and paraffin-embedded tissues were confirmed on haematoxylin and eosin-stained sections. The tissue sections (3-μm thick) were dewaxed, rehydrated and then immersed in methanol containing 0.3% hydrogen peroxide for 30 min to block endogenous peroxidase activity. Subsequently, the sections were heated in a pressure cooker filled with 10 mM ethylenediaminetetracetic acid (EDTA) buffer (pH 8.0) for 2 min. After cooling, the sections were incubated in 1% blocking serum for 30 min to reduce nonspecific binding. Primary anti-ZBTB20 polyclonal antibodies[39] were diluted 1:350 and incubated with the sections at 4°C overnight. Following incubation with biotinylated secondary antisera, the streptavidin-biotin complex/horseradish peroxidase was applied. Finally, the visualization signal was developed with diaminobenzidine (DAB) and the slides were counterstained in hematoxylin.

**Figure 1:**
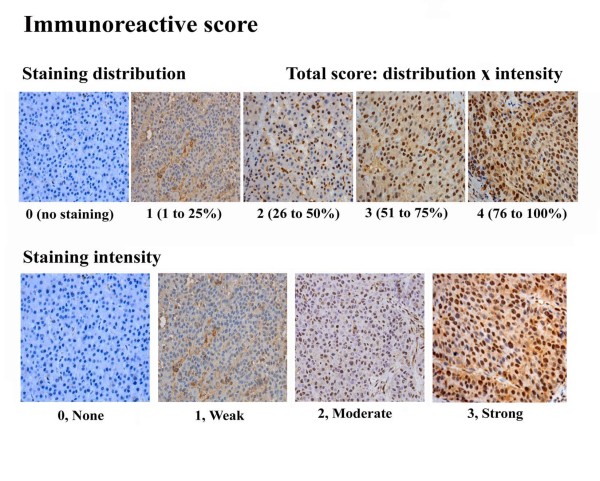
**Typical scored immunohistochemical staining of liver tissue specimens**. Formalin-fixed and paraffin-embedded tissues were incubated with primary anti-ZBTB20 polyclonal antibodies (diluted 1:350) at 4°C overnight. The visualization signal was developed with diaminobenzidine (DAB) and the slides were counterstained in hematoxylin. Total score was calculated from sub-scores of staining distribution (0-4) and intensity (0-3).

Stained sections were evaluated in a blinded manner without prior knowledge of the clinical information using the German immunoreactive score, Immuno-Reactive-Score (IRS). Briefly, the IRS assigns sub-scores for immunoreactive distribution (0-4) and intensity (0-3), then multiplies them to yield the IRS score. The percent positivity was scored as "0" (<5%), "1" (5-25%), "2" (25-50%), "3" (50-75%,), "4" (>75%). The staining intensity was score as "0" (no staining), "1" (weakly stained), "2" (moderately stained), and "3" (strongly stained). The final ZBTB20 expression score was calculated with the value of percent positivity score plus staining intensity score, which ranged from 0 to 12 (Figure 1). We estimated IRS by averaging the values in eight fields at ×400 magnification for each specimen. Intratumoral/peritumoral ZBTB20 expression was defined as follows: low expression (score 0-6/0-3) and high expression (score > 6/4-6). Immunohistochemical analysis and scoring were performed by 2 independent investigators.

### RNA extraction, cDNA synthesis and real-time RT-PCR

Total RNA was extracted from frozen tissue specimens (50-100 mg) using Trizol reagent (Invitrogen, Carlsbad, CA, USA), according to the manufacturer's protocol. The RNA was reverse transcribed using RT-PCR kits (Applied Biosystems, Foster City, CA, USA) with an oligo d (T) primer under standard conditions. Real-time ZBTB20 PCR amplification was performed in 1× Universal Master Mix (Applied Biosystems) with gene-specific primers and probe on the ABI Prism 7300 Sequence Detection System, according to the manufacturer's instructions. The 18s rRNA levels were quantified as internal control to normalize the expression of each gene. Each reaction was repeated independently at least three times. The following primers were used: ZBTB20, sense 5'- ATGCTAGAACGGAAGAAACCCA -3', antisense 5'-TGTGAGCGTGAGAGTTTGTCA -3';18s, sense 5'-GGGAGGTAGTGACGAAAAA T-3' and antisense 5'-ACCAACAAAATAGAACCGCG-3'. Relative expression levels of genes were calculated and expressed as 2^ΔCt^.

### Western blotting analysis

Western blotting was performed as previously described [41]. In brief, the tissues or cells were lysed in RIPA buffer, sonicated, and protein concentrations calculated by Bicinchoninic acid (BCA) (Sigma). About 60 μg protein samples were run on a 12% SDS-PAGE gel and transferred to a polyvinylidine difluoride membrane. Membranes were blocked for 1 h in Tris-buffered saline with 0.05% Tween 20 (TBST) containing 5% skim milk. The membrane was probed with ZBTB20 antibody (dilution, 1:2000) overnight followed by washing triplely with TBST for 5 min and incubated with an IRDye 800CW-conjugated secondary antibody. After being washed three times, the membrane was detected with LI-COR imaging system (LI-COR Biosciences). Mouse anti-human β-actin antibody (dilution, 1:1000; Santa Cruz) was applied as an internal control. The signal intensity of each band on scanned autoradiograms was analysed using LI-COR imaging system. We normalised each sample to the intensity of the β-actin band, and then estimated the value as an expression index.

### Follow-Up

Follow-up data were obtained for all the 102 patients with immunohistochemical analysis. The follow-up period was defined as the interval from the date of operation to the date of death or the last follow-up. Deaths from other causes were treated as censored cases. Recurrence and metastasis were diagnosed by clinical examination, AFP measurement, liver ultrasonography, and computed tomography (CT) scan. All patients were observed until December 2009, ranged from 29 to 103 months (median, 73 months). Overall survival (OS) was defined as the interval between the dates of surgery and death. Disease-free survival (DFS) was defined as the interval between the dates of surgery and recurrence; if recurrence was not diagnosed, patients were censored on the date of death or the last follow-up.

To determine factors influencing survival after operation, 13 conventional variables together with ZBTB20 expression were tested in 102 patients: age (≤50 years *vs *>50 years), gender, tumor size (≤50 mm *vs *>50 mm), serum AFP level (≤20 ng/mL *vs *>20 ng/mL), HBsAg (positive *vs *negative), Edmondson-Steiner grade (I-II *vs *III-IV), liver cirrhosis (yes *vs *no), tumor encapsulation (complete *vs *uncomplete *vs *no), vein invasion (yes *vs *no), tumour multiplicity (solitary *vs *multiple), TNM stage (I-II *vs *III), recurrence (yes *vs *no), metastasis (yes *vs *no), and ZBTB20 intratumoral/peritumoral protein expression level (high *vs *low).

### Statistical analysis

A paired-samples t-test was used to compare the mRNA and protein expression of ZBTB20 between HCC tumorous and the pair-matched non-tumor tissues samples. The Pearsontest or Fisher's exact test was used to analyze the relationship between ZBTB20 expression and the clinicopathologic features. The correlation between the expression of ZBTB20 and AFP was explored using Spearman's correlation analysis. Survival curves were calculated using the Kaplan-Meier method and compared by the log-rank test. The Cox proportional-hazard regression model was used for univariate and multivariate analyses to explore the effect of the clinicopathological variables and ZBTB20 expression on survival. SPSS 16.0 software (SPSS Inc., Chicago, IL, USA) was used for all statistical analyses and a *p *value < 0.05 was considered significant.

## Results

### Increased Expression Levels of ZBTB20 Messenger RNA and Protein in Hepatocellular Carcinoma

**Figure 2:**
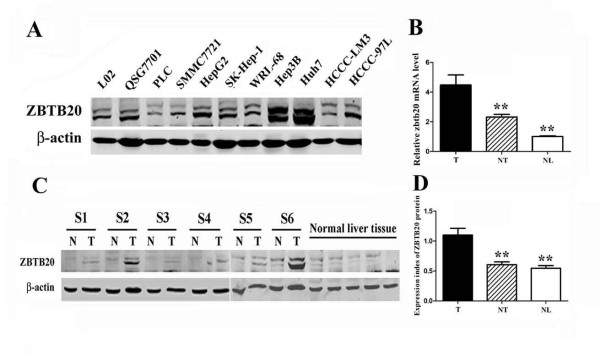
**ZBTB20 expression in HCC tissues and HCC cell lines**. A. Western blot analysis of ZBTB20 in HCC cell and normal liver cell lines. B. Evaluation of ZBTB20 mRNA in HCC. C. Western blot analysis of ZBTB20 in HCC and normal liver tissues. T Tumor tissue; NT non-tumor tissue; NL normal liver. D. Expression index of ZBTB20 protein. ZBTB20 protein expression level in HCC was significantly higher than those in NT (P < 0.001). The data are representative of three independent experiments.**: T compared with NT or NL, p <0.05.

Our previous work showed that ZBTB20 was developmentally up-regulated in liver and was a key repressor governing AFP gene transcription in postpartum liver, and may play an essential role in liver intrinsic functions by regulating genes such as P450 family members, glucose metabolism and the regulation of the somatotropic hormonal axis. To investigate the expression of ZBTB20 and its possible role in HCC, we first detected the protein expression in three human normal liver cell line (L02, QSG7701, WRL68) and 8 HCC cell lines (PLC/PRF/5, SMMC7721, HepG2, SK-Hep-1, Hep3B, Huh7, HCCCLM3, and HCCC97L). ZBTB20 was high expressed in most of the cells (Figure 2A, the whole image was shown in additional file 1). We further performed real-time RT-PCR for the transcriptional level of ZBTB20 from 50 frozen paired samples derived from patients who had undergone hepatectomy for histologically proven HCC. The mRNA level of ZBTB20 was significantly higher in 38 (76%) HCC tissues (tumour; T) compared with the pair-matched non-tumor tissues (NT). We calculated the copy number ratio of ZBTB20 mRNA/18s mRNA. This ratio was significantly higher in tumor samples than in NT samples (4.47 ± 2.71 versus 2.32 ± 0.64, *P *= 0.006) (Figure 2B).

Next, the protein level of ZBTB20 in T and NT specimens of 50 HCC cases was analyzed by western blot analysis. ZBTB20 signal was found to be positive in all T samples, and in 78% of NT samples. The intensity of each protein band was normalised against the expression of β-actin in each sample, and this ratio was treated as an expression index. Consistent with mRNA expression, the protein level of ZBTB20 in HCC tumor tissues was also significantly higher than that in NT samples (1.81-fold on average, *P *< 0.001) (Figure 2C).

### Immunohistochemistry Analysis of ZBTB20 Expression and its Relationship with the Clinicopathological Parameters

**Figure 3:**
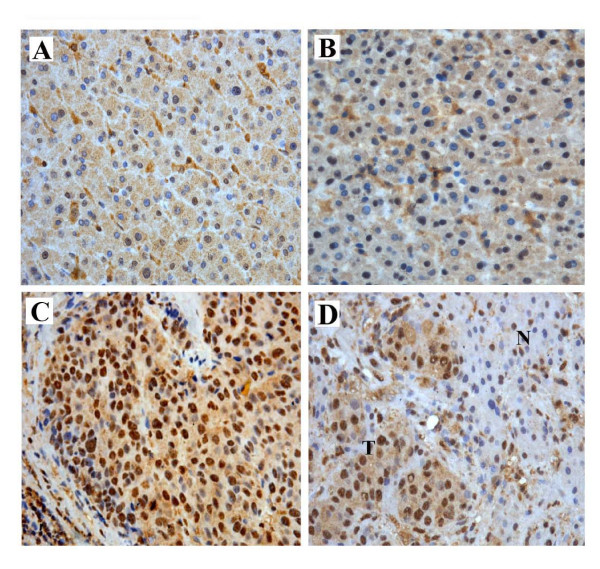
**Immunohistochemistry detection of ZBTB20 expression in normal liver tissue, cirrhotic liver tissue, HCC (T) and in noncancerous hepatic tissues (N)**. A. Normal liver tissue. B. Cirrhotic liver tissue. The expression of ZBTB20 was weakly positive in hepatocytes. C. HCC cells stained strongly positive with a large granular pattern. D The boundary area between the tumor and non-tumor lesion. Hepatocytes adjacent to HCC lesion were stained with a small granular pattern. Original magnification, ×200. T, tumor; N, Paired-nontumor tissue.

To investigate the effect and the prognostic value of ZBTB20, immunohistochemistry was used to assess the expression of ZBTB20 in the HCC tissues sections. It was showed that ZBTB20 staining was mainly in the nucleus of tumor cells, partly in the cytoplasm. ZBTB20 expression in normal hepatocytes from para-tumoral tissue of hemangiomas of liver and cirrhotic liver were negative or weakly positive (Figure 3A,B), while mainly moderate or strong positive in HCC tumors (Figure 3C). Overall, 42 of the 102 (41.2%) cases showed high expression (IRS over 6) ZBTB20 in the tumorous tissues, while 60 (58.8%) of the cases showed low expression (IRS 0-6). Generally, ZBTB20 density was significantly higher in intratumoral tissues than in peritumoral tissues (6.37 ± 3.17 versus 2.73 ± 1.68, *P *< 0.001) (Table 2). Additionally, a sharp contrast was often observed between infiltrative tumorous areas of positive staining and the adjacent nontumorous area of negative staining (Figure 3D).

**Table 2:** Average Density of Normal liver, Cirrhotic liver, Peritumoral and Intratumoral ZBTB20

staining	density			p
		
	mean	Standard deviation	range	
Normal liver	1	0.93	0-3	<0.001*
Cirrhotic liver	2.46	0.91	0-4	<0.001**
Peritumoral	2.73	1.68	0-6	<0.001***
Intratumoral	6.17	3.17	0-12	<0.001****

Abbreviations: * Normal liver tissue *vs *Cirrhostic liver tissue; ** Normal liver tissue *vs *Peritumoral; *** Normal liver tissue *vs *Intratumoral; ****Peritumoral *vs *Intratumoral.

We analysed the relationship between ZBTB20 protein expression and clinical features of HCC by establishing two groups with high and low ZBTB20 expression respectively based on the results of immunohistochemistry analysis. As shown in Table 3, intratumoral ZBTB20 density were positively correlated with vein invasion, recurrence and metastasis. Meanwhile, patients with high peritumoral ZBTB20 density were prone to recurrence. There was no significant association between ZBTB20 expression and the other clinical features, such as age, gender, tumor size, serum AFP level, HBsAg, Edmondson-Steiner grade, liver cirrhosis, tumor encapsulation, tumour multiplicity or TNM stage.

**Table 3:** Relationship Between Intratumoral and Peritumoral ZBTB20 and Clinicopathologic Features

	Intratumoral ZBTB20			Peritumoral ZBTB20		
				
Variable	High (n = 42, 41.2%)	Low (n = 60, 58.8%)	*P*	High (n = 42, 41.2%)	Low (n = 60, 58.8%)	*P*
**Age(years)**			0.523			1.0
≤50	19	31		20	30	
>50	23	29		22	30	
**Gender**			0.188			0.188
Male	36	45		36	45	
Female	6	15		6	15	
**Tumor size(mm)**			0.299			0.550
≤50	10	20		11	19	
>50	32	40		31	41	
**AFP(ng/ml)**						
≤20	10	16	0.745	11	15	0.892
>20	32	44		31	45	
**HBsAg**			0.302			0.106
Positive	38	50		39	49	
Negtive	4	10		3	11	
**Pathological stage**			0.396*			1.0*
I-II	1	5		2	4	
III-IV	41	55		40	56	
**Liver cirrhosis**			0.675			0.172
Yes	31	42		27	46	
No	11	18		15	14	
**Tumor encapsulation**			0.235			0.446
Complete	12	27		13	26	
Uncomplete	10	10		9	11	
No	20	23		20	23	
**Vein invasion**			0.016			0.161
Present	13	7		11	9	
Absent	29	53		31	51	
**Tumour multiplicity**			0.064			0.589
Solitary	25	46		28	43	
Multiple	17	14		14	17	
**TNM stage**			0.07			0.573
I-II	22	5		25	39	
III	20	55		17	21	
**Recurrence**			0.003			0.001
Yes	37	37		38	36	
No	5	23		4	24	
**Metastasis**			0.000			0.08
Yes	27	16		22	21	
No	15	44		20	39	

Abbreviations: HBsAg, hepatitis B s antigen.

*Twenty-five percent of all the cells have expected count less than 5; Fisher's exact test.

**Figure 4:**
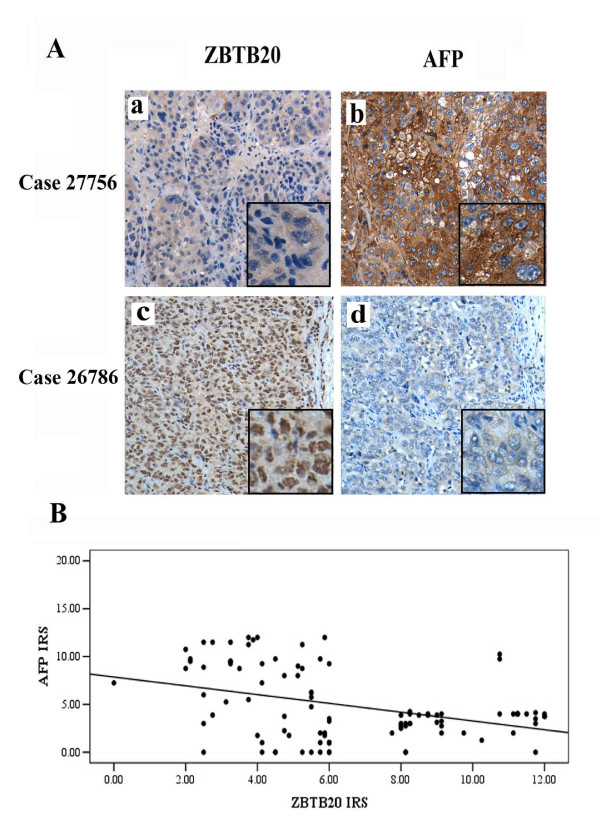
**Expression of ZBTB20 in HCC and correlation with AFP expression**. A. The consecutive slices of two samples immunostained by anti-ZBTB20 or anti-AFP antibody. a&b. Positive staining of AFP in HCC, while negative staining of ZBTB20; c&d. Positive staining of ZBTB20 in HCC, while negative staining of AFP. Magnification: ×100 & ×200. B. Scatter plot of the correlation of IRS of ZBTB20 with AFP. By Spearman's correlation analysis, IRS of ZBTB20 was negatively correlated to AFP (*r *= - 0.33, *P *= 0.001).

Our past work showed that ZBTB20 negatively regulated AFP [38]. However, in this work, no correlation between serum AFP level and tissue ZBTB20 protein expression was found. Considering the possible discrepancy of serum and liver tissue AFP level, we examined possible relationship between tissue AFP staining level and ZBTB20 expression level in HCCs, adopting Spearman's correlation analysis. In 102 samples, 61.8% cases (63/102) showed high IRS of ZBTB20 with low IRS of AFP; 29.4% cases (30/102) showed low IRS of ZBTB20 with high IRS of AFP; and 8.8% cases (9/102) showed almost the same level of IRS. As shown in Figure 4, the ZBTB20 level was negatively correlated with the staining degree of AFP in HCCs (*r *= -0.33, *P *= 0.001), while the serum AFP level had no significant correlation with the tissue AFP IRS (*P*= 0.538).

### Correlation between ZBTB20 Expression and Prognosis

**Figure 5:**
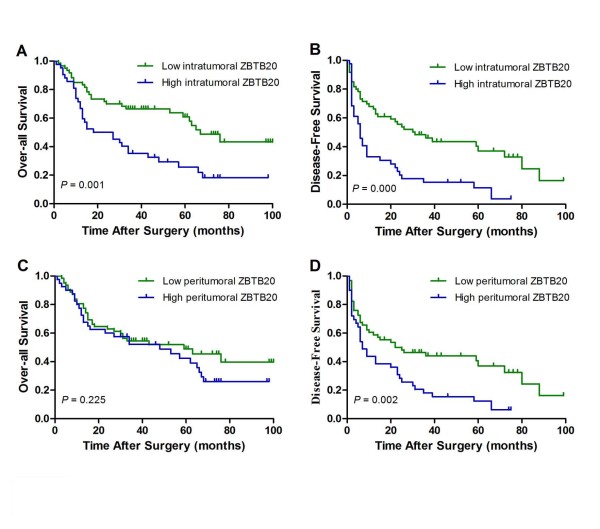
**Cumulative overall and disease-free survival curves of patients with high or low intratumoral or peritumoral features**. (A and B) Low intratumoral ZBTB20 was associated with prolonged overall and disease-free survival (*P *= 0.001 and *P*<0.001, respectively). (C and D) Low peritumoral ZBTB20 was associated with disease-free survival (*P *= 0.002) but not with overall survival(*P *= 0.225).

At the time of the last follow-up, 74 patients had tumor recurrence, and 59 patients had died. The 1-, 3-, and 5-year OS rates were 77.5%, 53.5%, and 45.9%, respectively; and the 1-, 3-, and 5-year DFS rates were 52.8%, 33.4%, and 26.5%, respectively. The median OS and DFS time were 18 months (95%CI, 1.06 months to 34.94 months) and 6 months (95%CI, 3.98 months to 8.01 months), respectively, for patients with high intratumoral ZBTB20 density (IRS over 6, as described in Materials and methods) and were statistically shorter than the median OS and DFS time for patients with low peritumoral ZBTB20 density [67 months (95%CI, 52.53 months to 81.47 months) and 31 months (95%CI, 13.89 months to 48.12 months), respectively (*P *= 0.001 and *P*<0.001, respectively; Figure 5A and 5B)]. Besides, patients with high peritumoral ZBTB20 had poor DFS (9 months *vs *22 months, *P *= 0.002; Figure 5D), whereas peritumoral ZBTB20 was not associated with OS (P = 0.225; Figure 5C)

Univariate Cox regression analyses determined that tumor size, serum AFP, TNM stage, tumor number, tumor encapsulation, vein invasion and intratumoral ZBTB20 expression were significantly associated with OS; while tumor size, HBsAg positive, TNM stage, tumor number, vein invasion and intratumoral/peritumoral ZBTB20 expression were associated with DFS. Furthermore, multivariate Cox regression analyses revealed that high intratumoral ZBTB20 was independent risk factor for OS (relative risk [RR] = 1.952, *P *= 0.016) and DFS (RR = 2.094, *P *= 0.003) (Table 4). Besides, tumor size large than 50 mm and vein invasion were also independent predictors for the OS of HCC patients (*P *= 0.006 and 0.086, respectively; Table 4), while the others failed to show this independence.

**Table 4:** Univariate and Multivariate Analyses of Factors Associated With Survival and Recurrence

Factor	OS				DFS			
	
		Multivariate				Multivariate		
				
	Univariate *P*	Hazard Ratio	95% CI	*P*	Univariate *P*	Hazard Ratio	95% CI	*P*
**Age**: ≤ 50 *v *>50 years	0.603			NA	0.782			NA
**Sex**: female *v *male	0.197			NA	0.254			NA
**Liver cirrhosis**: no *v *yes	0.932			NA	0.194			NA
**Tumor size**: ≤ 50 *v *>50 mm	0.001	2.727	1.326-5.605	0.006	0.001	2.175	1.22-3.875	0.008
AFP: ≤ 20 *v *>20 ng/ml	0.051			NA	0.070			NS
**HBsAg**: positive *v *negative	0.141			NA	0.042			NS
**TNM stage**: I-II *v *III	0.002			NS	υ0.0001	1.982	1.288-3.366	0.005
**Tumor number**: single *v *multiple	0.017			NS	0.0001			NS
**Tumor encapsulation: **complete *v *uncomplete *v *no	0.051			NA	0.065			NA
**Vein invasion**: yes *v *no	0.001	1.713	0.927-3.167	0.086	υ0.0001			NS
**Intratumoral ZBTB20**: low *v *high	0.001	1.952	1.134-3.359	0.016	0.0000	2.094	1.296-3.383	0.003
**Peritumoral ZBTB20**: low *v *high	0.225			NA	0.002			NS

Abbreviations: OS, overall survival; DFS, disease-free survival; NA, not adopted; HBsAg, hepatitis B s antigen; NS, not significant.

Using 12 months as the cutoff value, all of the recurrences were divided into early recurrence, which is mainly from intrahepatic metastasis, and late recurrence, which is usually a result of a multicentric new tumor [42]. Patients with higher intratumoral ZBTB20 density were prone to have an early recurrence compared with patients with low intratumoral ZBTB20 (7.72 ± 2.9 *vs *5.01 ± 2.86, *P*<0.001) (Table 5).

**Table 5:** Early Recurrence in HCC Patients

ZBTB20 density	Within 12 months		t value	*P*
			
	Recurrence (n = 51)	No recurrence (n = 51)		
Intratumoral	7.72 ± 2.9	5.01 ± 2.86	4.74	0.000
Peritumoral	2.98 ± 1.70	2.49 ± 1.63	1.484	0.141

## Discussion

In this study, we examined ZBTB20 expression profiles and its correlations with clinicopathologic parameters and prognosis in HCC. Our data revealed that both mRNA and protein levels of ZBTB20 were significantly higher in HCC tissues than in the corresponding non-tumor tissues and normal liver tissues.

We observed that increased expression of ZBTB20 in HCC was positively correlated with tumor vein invasion, recurrence and metastasis. Moreover, patients with high intratumoral ZBTB20 expression had significantly worse OS and DFS when compared with patients with low expression of ZBTB20. Multivariate analysis demonstrated that among the factors analyzed, intratumoral ZBTB20 expression was an independent prognostic factor for OS and DFS in patients with HCC. Meanwhile, tumor size, serum AFP, TNM stage, tumor number and vein invasion were all independent prognostic factors for OS; while tumor size, HBsAg positive, TNM stage, tumor number, vein invasion and peritumoral ZBTB20 expression were independent prognostic factors for DFS. These results clearly demonstrated that high ZBTB20 expression is associated with poor progression and unfavorable clinical outcome of HCC. This showed that high ZBTB20 expression in HCC is an indicator of poor prognosis.

Our earlier work demonstrated that ZBTB20 was a key repressor governing AFP gene transcription in postpartum liver. In this report, our data didn't show any relationship between ZBTB20 expression and serum AFP value. However, ZBTB20 and AFP expression, both of tumor tissue origin, were really inversely related (Figure 4). This was in keeping with the inconsistant relation between serum and tissue AFP level in HCC cases (unpublished data).

The close relationship between ZBTB20 expression and clinicopathological features predicted that ZBTB20 might boost carcinogenesis and tumor progression. Currently, several BTB/POZ-ZF proteins have been implicated in various cancers. For example, the BCL6 and PLZF proteins are causally involved in several types of B-cell lymphomas and acute promyelocytic leukaemia [6-11]. NAC-1 is significantly overexpressed in ovarian serous carcinomas and intense NAC-1 immunoreactivity in primary tumors predicts early recurrence [26] whereas transgenic mice that overexpress Pokemon develop aggressive tumours [15]. Among these proteins, their BTB/POZ domains play a critical role in carcinogenesis and tumor progression. Some researches suggested that targeting the BTB/POZ domain of BCL-6 caused apoptosis and cell cycle arrest, providing a novel approach for cancer therapy [7]. More recently, Cerchietti LC et al found that shock protein 90 (Hsp90) inhibitors selectively kill a subset of Diffuse Large B-cell Lymphomas (DLBCL) that are biologically dependent on the Bcl6 transcriptional repressor, through interrupting direct interaction between BCL-6 and Hsp90 followed by accelerated BCL-6 degradation [43].

## Conclusion

We demonstrated here, that ZBTB20 is overexpressed in a large proportion of patients with HCC and high ZBTB20 expression correlated with the disease progression and poor clinical outcome in HCC. Furthermore, ZBTB20 proved to be a risk factor for tumor recurrence and independent molecular marker of prognosis in HCC and may become a novel molecular target in the strategies for the prediction of tumor recurrence and prognosis or treatment of HCC.

## Abbreviations

HCC: Hepatocellular Carcinoma; BTB/POZ domain ZBTB20: Broad complex, tramtrack, and bric a brac; poxvirus and zinc finger; AFP: α feto-protein; IHC: immunohistochemistry; HBV: Hepatitis B virus; DAB: Diaminobenzidine; BSA: Bovine serum albumin; EDTA: Ethylenediaminetetracetic acid; IRS: Immuno-Reactive-Score; BCA: Bicinchoninic acid; PBS: Phosphate buffered solution; TBS(T): Triethanolamine-buffered saline(Tween-20); Tris: Tris(hydroxymethyl)aminomethane; SDS: Sodium dodecyl sulphate; OS: Overall survival; DFS: Disease-free survival.

## Competing interests

The authors declare that they have no competing interests.

## Authors' contributions

QW conducted the study and drafted the manuscript. YT conceived and designed the study. YR conducted the study and collected the clinical data. LD, ZX, LT, DC and WZ analyzed and interpreted the data. HH and HW provided the administrative support. All the authors have read and approved the final version of the manuscript.

## Pre-publication history

The pre-publication history for this paper can be accessed here:

http://www.biomedcentral.com/1471-2407/11/271/prepub

## Supplementary Material

Additional file 1 **Whole image of western blot showing ZBTB20 expression**. ZBTB20 expression was detected in 4 pairs of HCC specimens, 3 HCC cell lines (HepG2, PLC and Huh-7) and 293T cell line transiently transfected with ZBTB20-expressing plasmid as well as its negative control. Molecular Marker. N: Non-tumor T: Tumor. L:293T cell line transiently transfected with ZBTB20-expressing plasmid; R: 293T cell line transiently transfected with empty plasmid. Arrowhead indicates the ZBTB20 ladder. Click here for file
