# Supplementary figures and images for: Zinc finger protein ZBTB20 expression is increased in hepatocellular carcinoma and associated with poor prognosis
Source: BMC Cancer. 2011 Jun 25;11:271. doi: 10.1186/1471-2407-11-271 (PMC3145616; doi:10.1186/1471-2407-11-271)

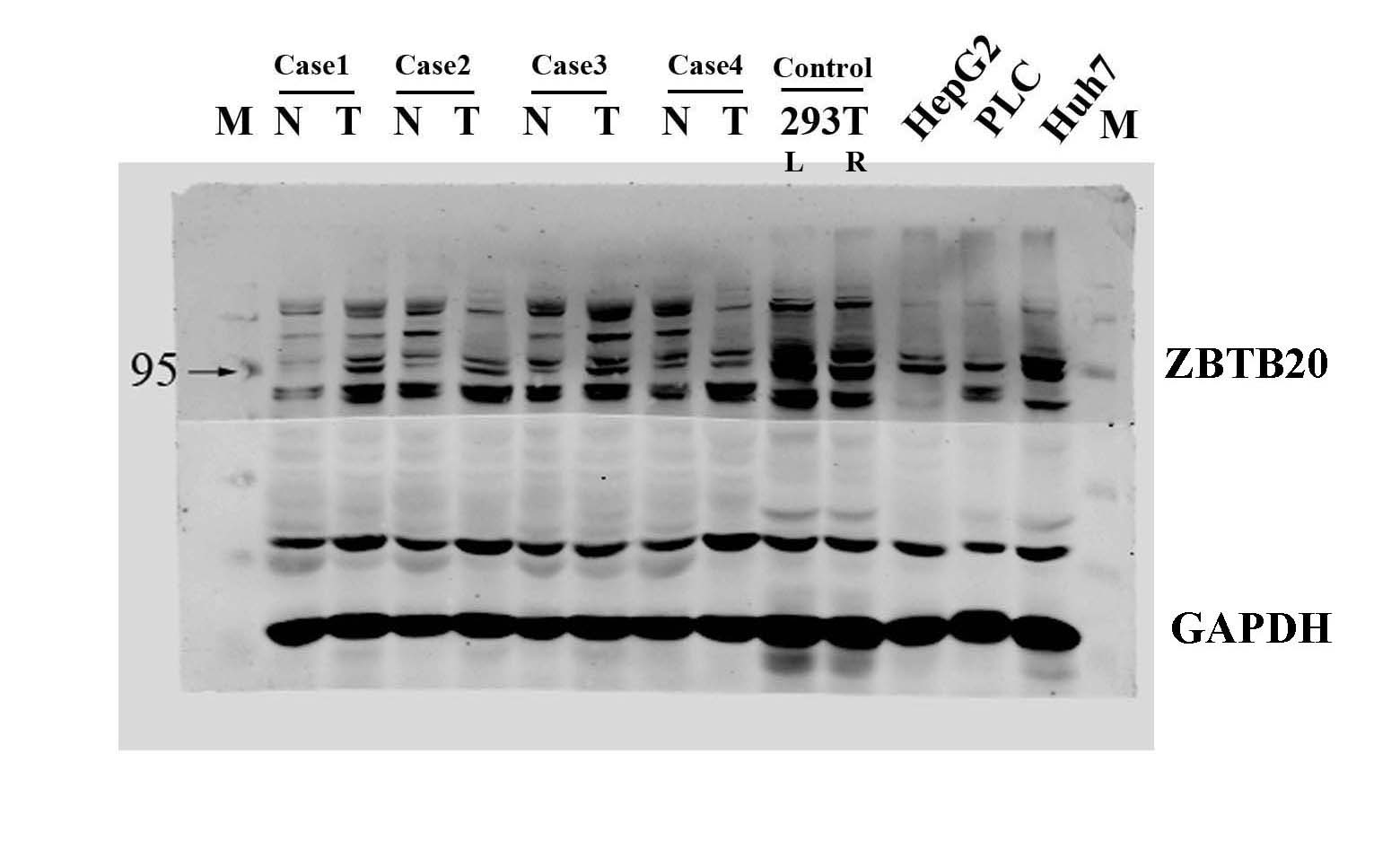

Supplement: Additional file 1 — Whole image of western blot showing ZBTB20 expression. ZBTB20 expression was detected in 4 pairs of HCC specimens, 3 HCC cell lines (HepG2, PLC and Huh-7) and 293T cell line transiently transfected with ZBTB20-expressing plasmid as well as its negative control. Molecular Marker. N: Non-tumor T: Tumor. L:293T cell line transiently transfected with ZBTB20-expressing plasmid; R: 293T cell line transiently transfected with empty plasmid. Arrowhead indicates the ZBTB20 ladder. [file 1471-2407-11-271-S1.JPEG]
